# Wavelength-dependent photodissociation of iodomethylbutane

**DOI:** 10.1038/s41598-025-04905-5

**Published:** 2025-06-20

**Authors:** Valerija Music, Felix Allum, Ludger Inhester, Philipp Schmidt, Rebecca Boll, Thomas M. Baumann, Günter Brenner, Mark Brouard, Michael Burt, Philipp V. Demekhin, Simon Dörner, Arno Ehresmann, Andreas Galler, Patrik Grychtol, David Heathcote, Denis Kargin, Mats Larsson, Jason W. L. Lee, Zheng Li, Bastian Manschwetus, Lutz Marder, Robert Mason, Michael Meyer, Huda Otto, Christopher Passow, Rudolf Pietschnig, Daniel Ramm, Daniel Rolles, Kaja Schubert, Lucas Schwob, Richard D. Thomas, Claire Vallance, Igor Vidanovic, Clemens von Korff Schmising, René Wagner, Vitali Zhaunerchyk, Peter Walter, Sadia Bari, Benjamin Erk, Markus Ilchen

**Affiliations:** 1 Department of Physics, Universität Hamburg, 22607 Hamburg, Germany; 2 Institut für Physik und CINSaT, Universität Kassel, 34132 Kassel, Germany; 3 European X-Ray Free-Electron Laser Facility, 22869 Schenefeld, Germany; 4 Stanford PULSE Institute, SLAC National Accelerator Laboratory, Menlo Park, CA 94305 USA; 5 The Chemistry Research Laboratory, Department of Chemistry, University of Oxford, Oxford, OX1 3TA UK; 6 Center for Free-Electron Laser Science CFEL, Deutsches Elektronen-Synchrotron DESY, Notkestr. 85, 22607 Hamburg, Germany; 7 Deutsches Elektronen-Synchrotron DESY, Notkestr. 85, 22607 Hamburg, Germany; 8 Institut für Chemie, Universität Kassel, Heinrich-Plett-Straße 40, 34132 Kassel, Germany; 9 Stockholm University, AlbaNova University Center, 114 21 Stockholm, Sweden; 10 State Key Laboratory for Mesoscopic Physics, School of Physics, Peking University, Beijing, 100871 China; 11 Collaborative Innovation Center of Extreme Optics, Shanxi University, Taiyuan, 030006 Shanxi China; 12 Kansas State University, 1228 N 17th St, Manhattan, KS 66506 USA; 13 Max Born Institute, Max-Born-Straße 2A, 12489 Berlin, Germany; 14 University of Gothenburg, 405 30 Gothenburg, Sweden; 15 SLAC National Accelerator Laboratory, 2575 Sand Hill Road, Menlo Park, CA 94025 USA; 16 TAU Systems, Austin, TX USA

**Keywords:** Chemical physics, Excited states, Atomic and molecular interactions with photons

## Abstract

Ultrashort XUV pulses of the Free-Electron-LASer in Hamburg (FLASH) were used to investigate laser-induced fragmentation patterns of the prototypical chiral molecule 1-iodo-2-methyl-butane ($\hbox {C}_5$ $\hbox {H}_{11}$I) in a pump-probe scheme. Ion velocity-map images and mass spectra of optical-laser-induced fragmentation were obtained for subsequent FEL exposure with photon energies of 63 eV and 75 eV. These energies specifically address the iodine 4d edge of neutral and singly charged iodine, respectively. The presented ion spectra for two optical pump-laser wavelengths, i.e., 800 nm and 267 nm, reveal substantially different cationic fragment yields in dependence on the wavelength and intensity. For the case of 800-nm-initiated fragmentation, the molecule dissociates notably slower than for the 267 nm pump. The results underscore the importance of considering optical-laser wavelength and intensity in the dissociation dynamics of this prototypical chiral molecule that is a promising candidate for future studies of its asymmetric nature.

## Introduction

Photodissociation is an important restructuring process of matter with a broad interest in the biological, chemical, and physical sciences. The time-resolved investigation of photodissociation with element- or ‘site’-specificity provides unique insight into the different dynamics and pathways of e.g., molecular fragmentation, the underlying processes of radiation damage, chemical bond-breaking, and light-matter interaction in general^1^. These processes evolve to a significant extent on femtosecond timescales and are therefore only directly accessible in the time domain via pulsed light sources with pulse durations of the order of the dynamics of interest. Noteworthy in this regard are, among others, femtosecond optical lasers (OLs) and short-wavelength free-electron lasers (FELs)^2^, the latter enabling distinct element specificity due to the availability of short wavelengths. In fact, (X)FELs can provide a broad range of photon energies from the extreme ultraviolet (XUV) to hard X-ray pulses with femtosecond to attosecond duration and up to the mJ-level of pulse energy, thus opening a variety of opportunities to investigate nonlinear and ultrafast processes^1,3,4^.

**Fig. 1:**
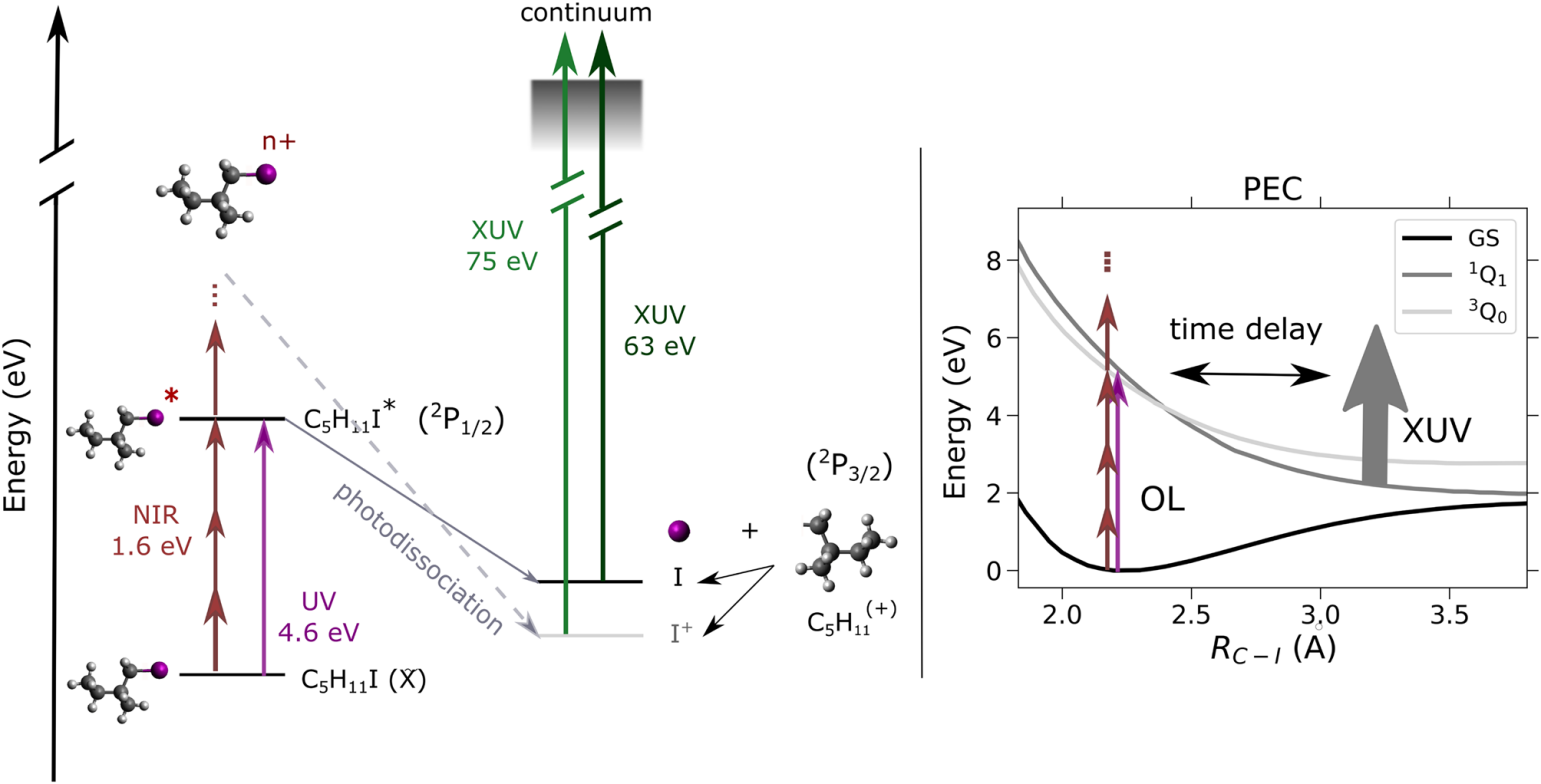
Schematic representation of the pump-probe experiment on 1-iodo-2-methyl-butane. The optical laser pulses photoexcite the molecule, either via one photon (4.6 eV, violet arrow) or multiphoton (1.6 eV, maroon arrows) absorption. Via the excited states, the molecule dissociates along the C–I bond involving either neutral or singly charged iodine which was subsequently probed via 4d photoionization using ultrashort XUV pulses. The potential energy curves displayed on the right were calculated via $\Delta$CASSCF for the ground state and the two excited states $^1$ $\hbox {Q}_1$ and $^3$ $\hbox {Q}_0$ resulting from the neutral dissociation along the C–I bond.

Here, we report on the investigation of OL-induced fragmentation processes of isolated 1-iodo-2-methyl-butane molecules ($\hbox {C}_{5}$ $\hbox {H}_{11}$I) resulting from interaction with ultraviolet (UV) or near-infrared (NIR) pulses with a wavelength (photon energy) of 267 nm (4.6 eV) and 800 nm (1.6 eV), respectively. The molecule as a prototypical chiral molecular system is interesting in several regards, such as its easy experimental accessibility and the presence of a heavy ‘marker’ atom outside its stereocenter as a possible observer, i.e. electron emission, site^5–9^. For both OL wavelengths, intensity-dependent ion yields have been obtained in order to identify different regimes of fragmentation into neutral or charged fragments. The respective changes have furthermore been investigated for their ultrafast time evolution using two XUV-FEL photon energies at 63 eV and 75 eV. These energies were chosen to predominantly ionize the 4d edges of the neutral and additionally singly-charged iodine in the fragments, respectively, resulting in similar electron kinetic energies (see Fig. 1).

Notably, when neutral iodine atoms are formed, the binding energies of the 4d electrons in iodine change from their values in the molecular environment $\approx$ 56.5 eV (4$\hbox {d}_{5/2}$) and $\approx$ 58 eV (4$\hbox {d}_{3/2}$), to isolated atomic iodine by $\approx +1.7$ eV^10^. For the fragmentation forming singly-charged iodine cations, we calculate a binding energy increase of the iodine 4d-electrons of about 12 eV from the neutral molecule to the isolated iodine cation via $\Delta$CASSCF modeling (averaged over the two spin contributions)^11^. We establish selected fragmentation benchmarks for this prototypical chiral molecule with the future aim to investigate time-resolved photoelectron circular dichroism (PECD) phenomena^12–16^, the latter being the reason for the choice of the same electron kinetic energies in neutral and ionic iodine.

## Experiment

The experiments were conducted at the BL1 beamline of the Free-Electron LASer in Hamburg (FLASH)^17,18^. A double-sided velocity-map-imaging (VMI) spectrometer which is a part of the CAMP endstation^19^ was used to measure cations produced by photoionization of the chiral iodoalkane molecule $\hbox {C}_{5}$ $\hbox {H}_{11}$I and its fragments, with a predominant scope of addressing the I 4$\hbox {d}_{3/2}$ and I 4$\hbox {d}_{5/2}$ vacancy states in different charge environments, thus involving different ionization potentials. At 63 eV the photoionization cross section of the iodine 4d is approximately 3 Mbarn^20^ and for 75 eV is approximately 13 Mbarn^20,21^). These cross sections are similar to the sum of the photoionization cross sections of the remaining constituents.

In the center of the double-sided VMI spectrometer, the $\hbox {C}_{5}$ $\hbox {H}_{11}$I molecular jet, discussed further below, was crossed with the UV or NIR pump laser, inducing the molecules’ photo-excitation and/or -ionization. Both linearly polarized ’pump’-laser pulses were generated using an 800 nm Ti:Sapphire laser^22^ which propagates at a small angle of 1.5 $^\circ$ with respect to the FEL- and quasi-perpendicular to the molecular beam. The 800 nm (1.6 eV) fundamental wavelength was delivered at 10 Hz with an approximate pulse duration of 70 fs (FWHM). These pulses with a maximum energy of 1.8 mJ were focused to a spot size with a FWHM diameter on the order of 60 µm $\pm ~20$ µm. The 267 nm (4.6 eV) pulses were created by third-harmonic generation using a Beta Barium Borate (BBO) crystal^23^ with a maximum pulse energy of 176 µJ, a focus diameter of about 100 µm $\pm 40$ µm, and a pulse duration of about 150 fs $\pm ~30$ fs. redIt is noteworthy that the effective foci that contribute to the ionization processes under investigation are significantly smaller than the above-stated diameters due to the multiphoton-ionization nature of the studied processes. Consequently, this effective focal volume is even smaller for the NIR than for the UV which could, in principle, lead to a reduced contrast between pumped and unpumped target. The following intensity ranges were covered by the UV and NIR pulses: $(0.4-5.2)\cdot 10^{12}$ W/$\hbox {cm}^2$ and $(1.3-4.1)\cdot 10^{14}$ W/$\hbox {cm}^2$. These values correspond to pulse energies between 0.05 µJ to 87.8 µJ and 0.36 mJ to 1.16 mJ, respectively.

The time-delayed circularly polarized^24^ XUV pulses were generated by the FLASH1 FEL with a repetition rate of 10 Hz (single bunch mode) limited by the maximum achievable synchronization rate between OL and FEL under the given conditions. The circular polarization allows, in principle, for chiroptically sensitive measurements, however, for the here presented findings this is not relevant. The pulse energies of the XUV pulses were chosen to be relatively low in order to minimize XUV-driven nonlinear effects on the sample and were 10 µJ for the setting using 63 eV and 20 µJ for 75 eV. They were focused into a spot with a diameter of about 10 µm via Kirkpatrick-Baez optics, and the pulse duration was about 80 fs for both settings. The FEL focus size was chosen to be much smaller than the OL focus size in order to ensure optimum pump-probe contrast. The FEL pulse duration was retrieved via electron-beam diagnostics called ‘LOLA’^25,26^. The pulse energy and timing fluctuations were recorded by the FLASH Gas Monitor Detector (GMD)^27^ and Bunch Arrival Monitor^28^, respectively. The jitter between the XUV and the optical laser is thus determined to be about 20 fs which is included in the described uncertainties.

**Fig. 2:**
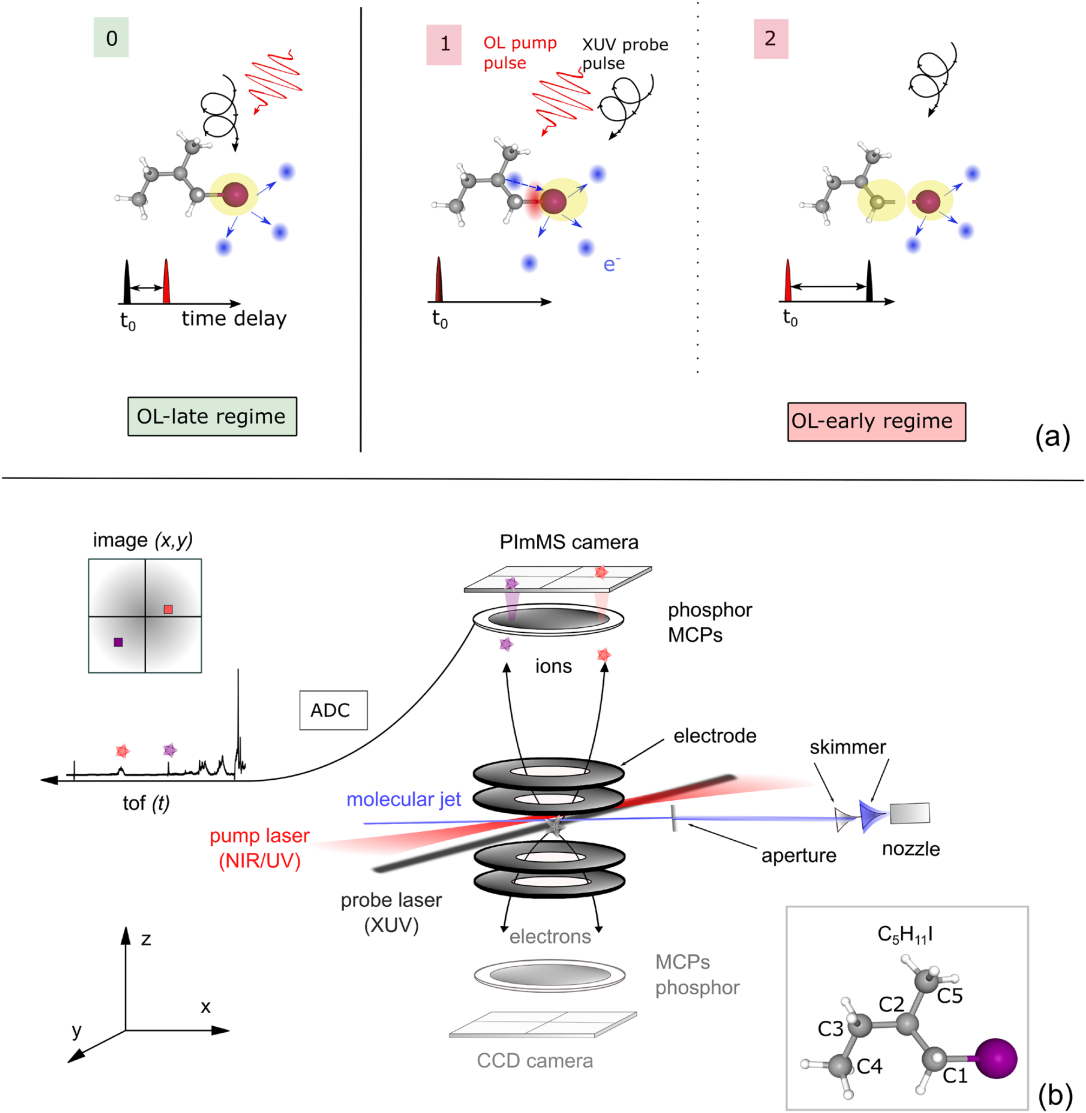
(**a**) Sketch of molecular dissociation for two different regimes. The temporal overlap of the OL and XUV pulses is defined as time zero ($\hbox {t}_0$)^10^. In green, the OL-late regime is displayed. Here, the molecule is first illuminated by XUV and then by OL pulses (0). Secondly in red, the OL-early regime is displayed. Here, OL-pump pulses initiate a photodissociation of the molecule and time-delayed XUV pulses probe the iodine at two exemplary times (1) and (2). (**b**) Sketch of the experimental set-up: The injection of the molecules is depicted via the blue line system starting at the middle right. The skeletal of the molecule with annotated carbon atoms is visualized on the lower right. In the VMI spectrometer indicated by the round plates, the molecules are intersected by an optical pump laser (red) and the time-delayed FEL (black). The created charged particles, cations and electrons, were guided in z-direction and imaged on position-sensitive detectors (laboratory frame orientation indicated on the lower left). The polarization of the optical laser is in the x–y plane for the optical laser and in the x–z plane for the circularly polarized XUV. The ToF readout provides information about the different arrival times and thus mass/charge spectra. In the scope of this work, solely the cation data is analyzed and presented.

The liquid molecular sample (99% purity, Merck KGaA, Darmstadt, Germany and also synthesized in our laboratories in Kassel, Germany, according to established literature procedures^29^) was stored in a cylinder and was heated up to 80 $^{\circ }$C with a positive gradient towards the jet nozzle in order to prevent clogging of the sample. The evaporated sample was seeded in a helium carrier gas at 1 bar backing pressure and transported into a heated sample-delivery line ending with a jet nozzle of a diameter of 30 µm. The continuous molecular beam passed through two skimmers with orifice diameters of 150 µm and 350 µm, in this sequence. Finally, the molecular jet passes through an aperture of 4 mm opening for differential pumping, placed approximately 35 cm away from the interaction volume. As sketched in Fig. 2b, this doubly skimmed continuous supersonic molecular jet (in blue) was injected into the interaction region, and intersected by the OL (in red), and time-delayed XUV pulses of the FEL (in black).

The voltages on the electrodes of the double-sided VMI were chosen such that charged particles with the same initial velocity were focused along the z-axis into the same point on the detector^19,30^. The polarization of the optical-laser photons is in the x–y plane whereas the circularly polarized XUV is polarized in the x–z plane, as illustrated on the bottom left of the figure. On both spectrometer sides, the particles hit 80 mm Chevron-stacked Multi-Channel Plates (MCPs) producing electron avalanches which were accelerated to P47 phosphor screens. For the positively-charged ions as subject of this work, a Pixel Imaging Mass Spectrometry (PImMS)^31^ camera with 324 $\times$ 324 pixels was used as read-out of the screen illumination. The signals were furthermore independently monitored by a capacitive outcoupling of the MCP’s current, thus providing time-of-flight information (*t*) that can be converted to mass spectra. Contributions of stray-light-induced background electrons were reduced by fast HV switches (Behlke HTS31-GSM) that defined a temporal window of operation for the MCPs. Part of the electron data, concerning changes in the I 4d binding energy induced by single-photon 267 nm excitation, was published previously^10^.

Figure 2a shows the two different time-delay regimes that were used within this work and are depicted as OL-late (in green) and OL-early (in red) regime. Time zero ($\hbox {t}_0$) defines the temporal overlap of the OL and XUV pulses. Firstly, in the OL-late regime in green (0) the molecule is illuminated by XUV pulses and afterward by the OL pulses. Secondly, in the OL-early regime, OL-pump pulses initiate the photodissociation of the molecule. The time-delayed XUV pulses subsequently probe the iodine exemplarily at two different times^32^: (1) XUV pulses arrive shortly after OL pulses. Here, the XUV pulses ionize the iodine and create charges, which can still be redistributed over the dissociating molecule^5,33^. (2) At larger time delays between the two pulses, the molecule is dissociated, thus the charge transfer channel is closed. However, the Coulomb interaction between the fragments is still evolving. Ejected electrons are visualized in blue and the charge is indicated by a yellow surrounding.

## Results and discussion

### Intensity dependence of the pump process for UV and NIR

The cation yields after dissociation of 1-iodo-2-methyl-butane induced by two OL wavelengths and at various OL intensities without XUV probe pulses are investigated.

**Fig. 3:**
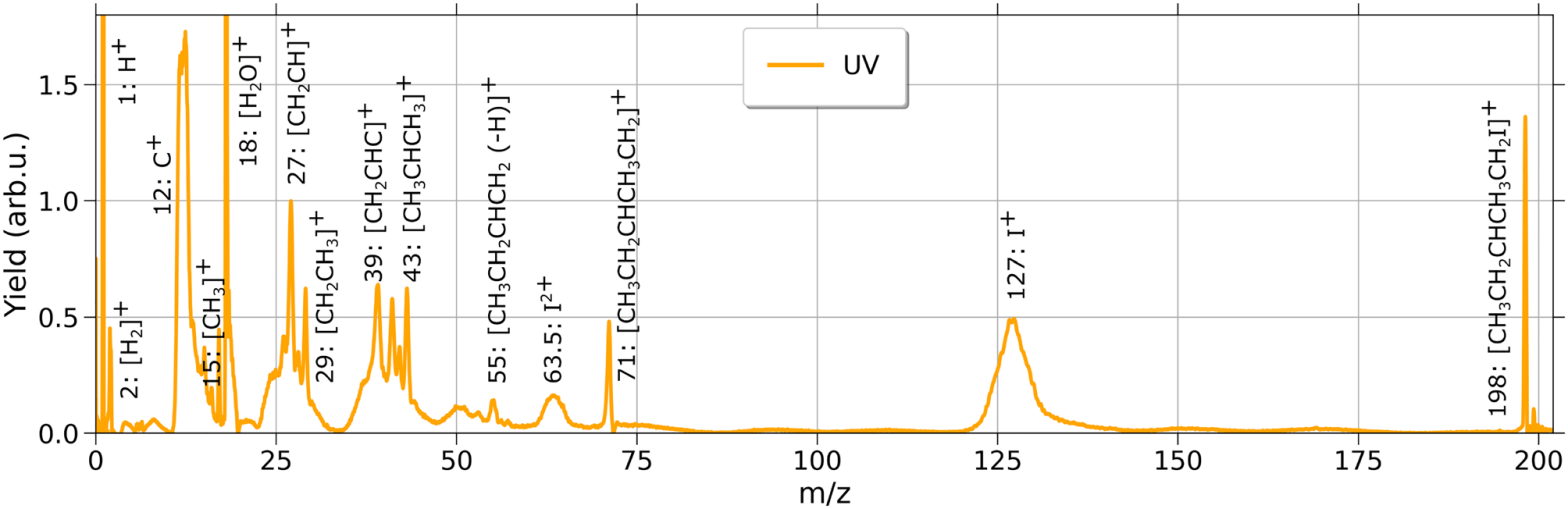
Mass spectrum for one exemplary UV (267 nm) laser intensity of 5.2 $\times 10^{12}$ W/$\hbox {cm}^2$. The most prominent peaks are labeled. The yield of the cations in arbitrary units, plotted on the y-axis and normalized to the peak *m*/*z*=27.

**Fig. 4:**
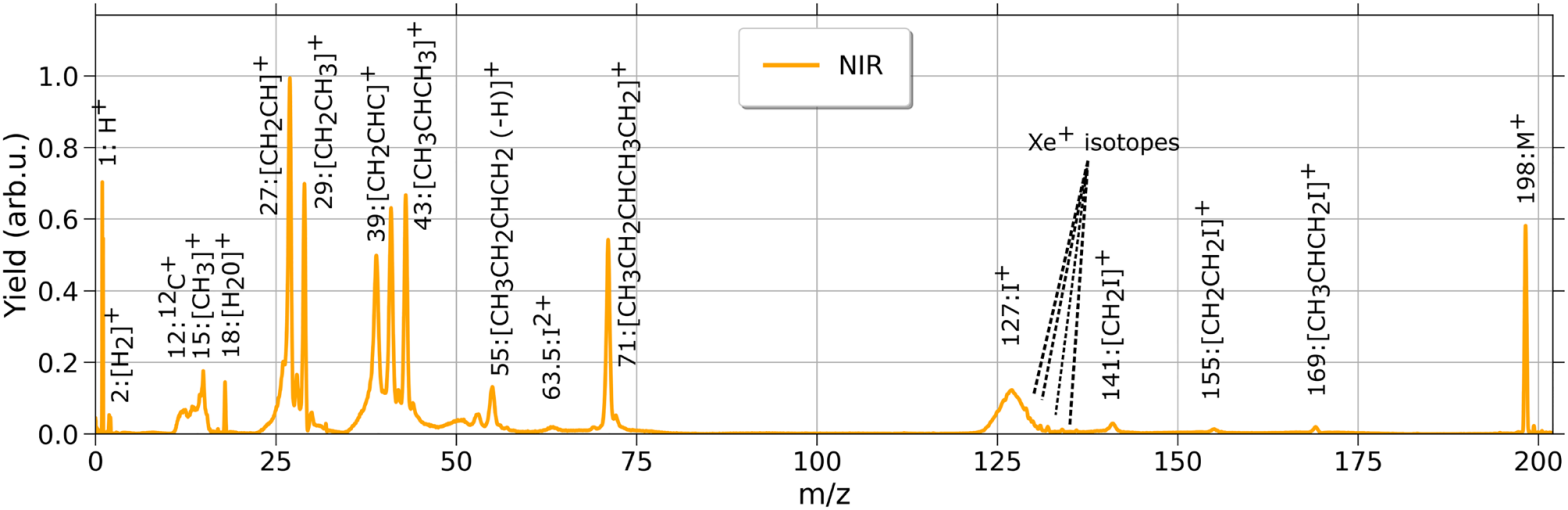
Mass spectrum for one exemplary NIR (800 nm) laser intensity 4.1 $\times 10^{14}$ W/$\hbox {cm}^2$. Plotted are mass over charges against their yield in arbitrary units, normalized to the highest peak *m*/*z*=27. The iodine peak is partially overlapped by residual xenon, resulting from a calibration data set taken previously.

The mass spectra obtained via UV or NIR pulses are illustrated in Figs. 3 and 4 for a selected exemplary intensity value, respectively. The spectra reveal distinctly different fragmentation patterns, as expected due to the differently triggered dissociation dynamics at different irradiation levels (see Fig. 1).

A variety of ionic fragments as well as the ionic intact molecule at *m*/*z*=198 are depicted to give an overview over exemplary fragmentation distributions.

In comparison, in the NIR mass spectrum (Fig. 4) cations with the highest yield are the parent cation $\hbox {C}{_5}$ $\hbox {H}{_{11}}$I${^+}$ at *m*/*z*=198, $\hbox {C}{_5}$H${_{11}}$ ${^+}$ at *m*/*z*=71 and various alkyl groups $\hbox {C}_n$ $\hbox {H}_x^+$ (n = 2-4 and x = 3, 5, and 7), which constitute the *m*/*z* from 27 to 55.

Thus, for these NIR pulses, multiple fragmentation channels were populated, resulting in more ionic channels, and yielding several pathways with comparable abundance. Furthermore, the multiphoton dissociation induced by the NIR pulses yields three additional peaks which result from predominantly dissociating the molecule at a C–C and not the C–I bond. This can occur between the second and third carbon atom ($\hbox {CH}_3$ $\hbox {CHCH}_2$I, *m*/*z*=169), the second and third plus second and fifth carbon atom ($\hbox {CH}_2$ $\hbox {CH}_2$ $\hbox {I}^+$, *m*/*z*=155) or the first and second carbon atom ($\hbox {CH}_2$ $\hbox {I}^+$, *m*/*z*=141) (see illustration shown in the right bottom corner of Fig. 2b).

**Fig. 5:**
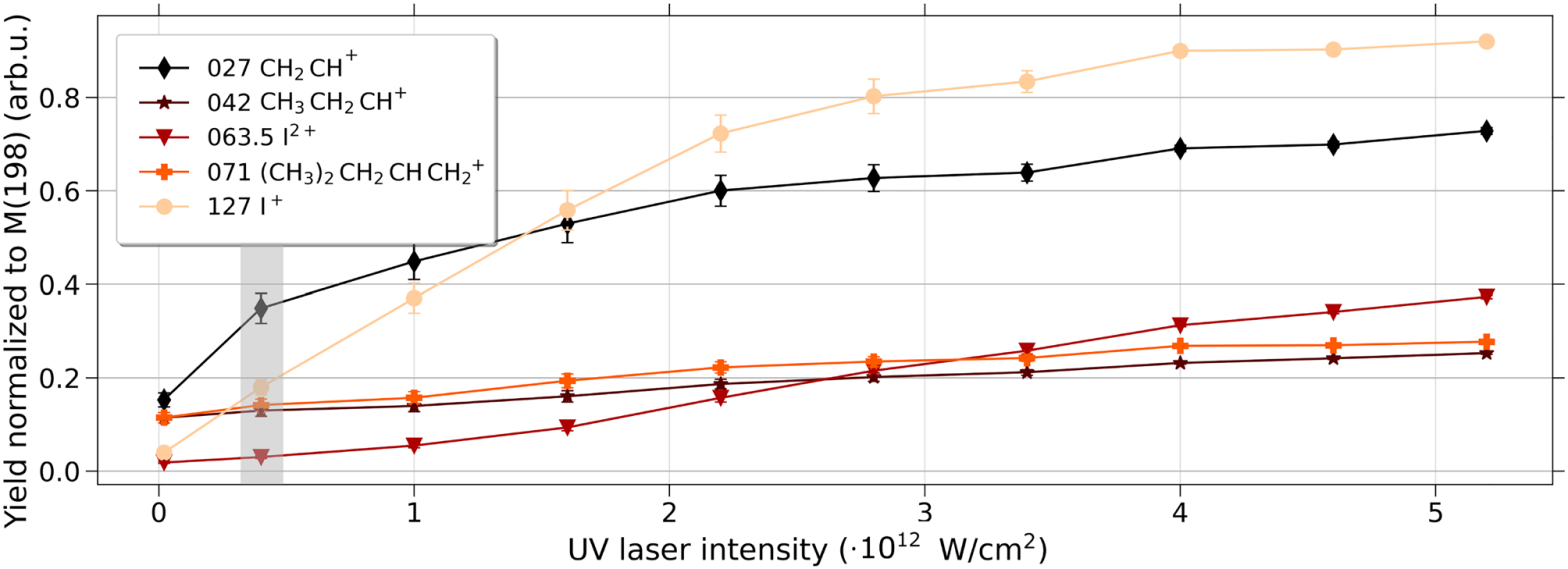
Yield of specific cationic fragments as a function of the UV laser intensity. Each value was normalized to the parent ion yield. Plotted are the mass over charges 27, 42, 63.5, 71 and 127. The lower intensity regime of the UV pulses, used for the below-presented UV-pump XUV-probe data, is shaded in light gray.

**Fig. 6:**
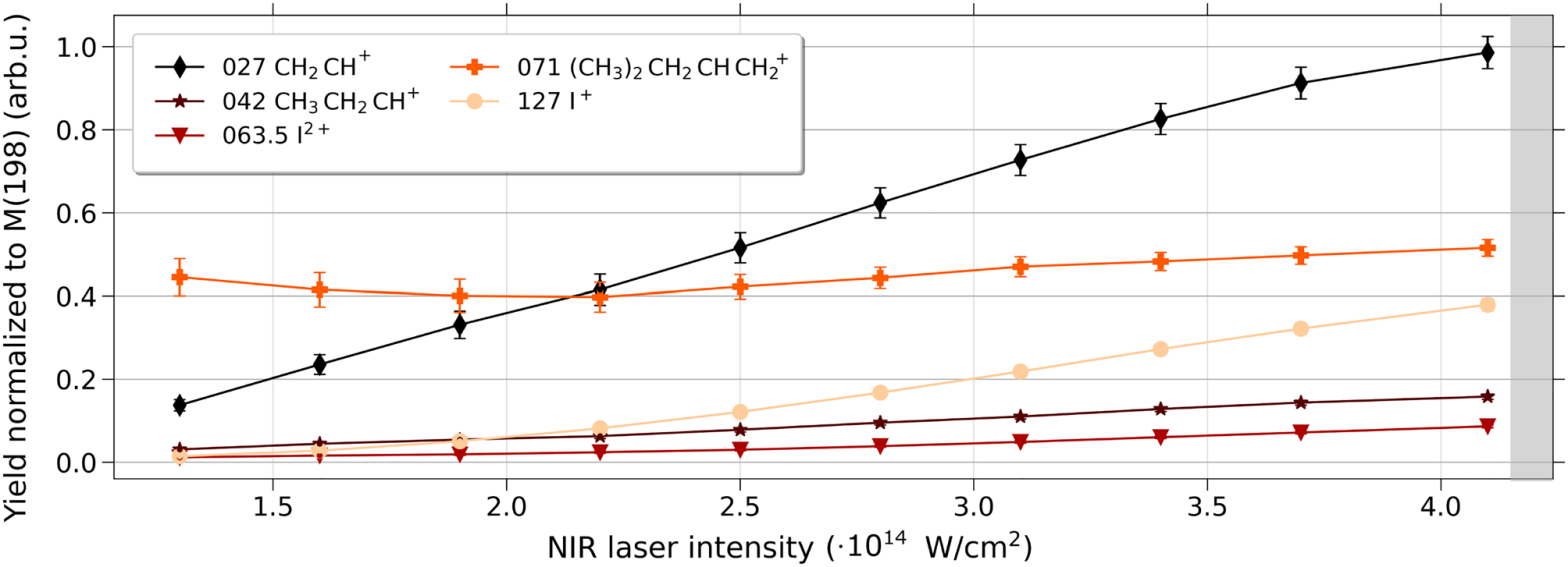
Yield of specific cationic fragments as a function of the NIR laser intensity. The yield is normalized to the yield of the parent cation with *m*/*z*=198. The higher intensity regime of the NIR pulses used, for the below-presented NIR-pump XUV-probe data, is shaded in light gray.

Figures 5 and 6 depict the yield of selected cations as a function of the laser intensity. Each cation yield was normalized to the yield of the parent cation. The analysis was performed by integrating the area under each mass peak using single Gaussian fitting. Represented cations in the two figures are $\hbox {I}^+$ (*m*/*z*=127), $\hbox {C}_5$ $\hbox {H}_{11}$ $^+$ (*m*/*z*=71), $\hbox {I}^{2+}$ (*m*/*z*=63.5), $\hbox {C}_3$ $\hbox {H}_7$ $^+$ (*m*/*z*=42), and $\hbox {C}_2$ $\hbox {H}_3$ $^+$ (*m*/*z*=27). The changing yields, here with emphasis on the $\hbox {I}^{+}$, are an indication of different charge-up and dissociation pathways, which are the basis for the respective intensity choice for the time-resolving experiments presented below. The other fragments shown are a benchmark for reference.

As shown in Fig. 5, the yield of the singly-charged iodine fragment (light yellow dots) increases drastically as a function of the UV laser intensity and is quite dominantly produced at higher intensities. Since the goal of the UV-part of the experiment was to trigger neutral dissociation of the iodine atom the marked intensity slightly above the threshold of the appearance of the $\hbox {I}^{+}$ fragment was chosen in this case (UV intensity of 0.4 $\times 10^{12}$ $\hbox {W}/\hbox {cm}^2$, see gray area on the left of Fig. 5)^34,35^.

In contrast, as shown in Fig. 6, the overall yield of the singly charged iodine in the investigated range of NIR intensities shows a more moderate increase towards higher intensities, and the total yield is smaller compared to other fragments. To ensure a high number of singly-charged iodine fragments for probing via the XUV pulses, a high NIR intensity of 4.3 $\times 10^{14}$ W/$\hbox {cm}^2$ was chosen to be used for the respective pump-probe experiments (see gray area on the right of Fig. 6).

### Time-resolved results

**Fig. 7:**
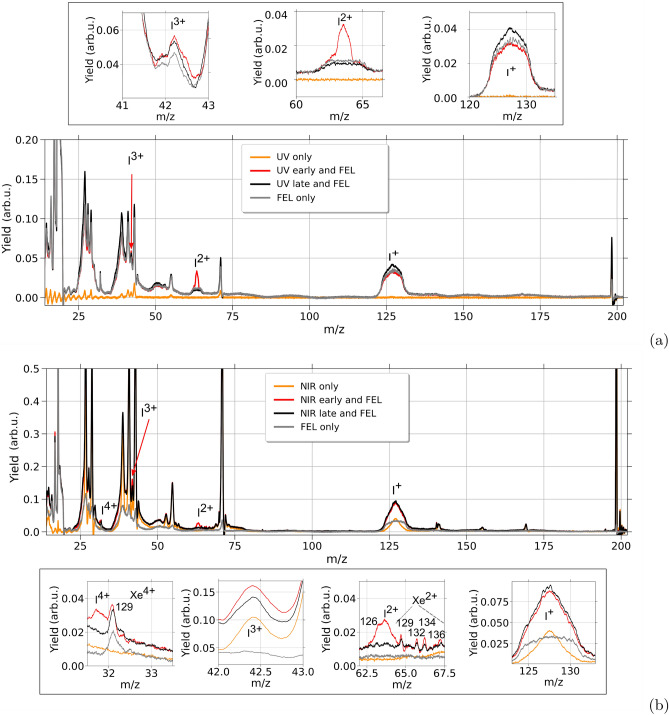
Mass spectra of 1-iodo-2-methyl-butane for different pump and probe schemes, (**a**) for the UV and (**b**) for the NIR pulses with an intensity of 0.4 $\times 10^{12}$ W/$\hbox {cm}^2$ and 4.3 $\times 10^{14}$ W/$\hbox {cm}^2$, respectively. Compared are mass spectra resulting from the OL (orange), from the XUV-FEL ionization (gray), from OL-pump pulses 500 fs (UV)/2 ps (NIR) before the XUV-probe pulses (red, OL-early regime) and lastly from the probe pulses 500 fs (UV)/1 ps (NIR) before the pump pulses (black, OL-late regime). Enlarged views are plotted for ($\hbox {I}^{4+}$), $\hbox {I}^{3+}$, $\hbox {I}^{2+}$, and $\hbox {I}^{+}$ fragments. Short cation flight times are subject to ringing due to electronic feedback from the pulsing of the high voltage of the electron detector. Mass-to-charge ratios *m*/*z*=13 are therefore not analyzed. Xenon peaks result from residual gas of a previous calibration measurement. The FEL data was normalized by the GMD and the number of shots. A quantitative comparison between (**a**) and (**b**) cannot be provided by this data due to the different experimental conditions.

The goal of the time-resolving investigations was to distinguish between Coulomb-driven and neutrally dissociative fragmentation for NIR-pump and UV-pump experiments, respectively, at specific intensity choices based on the previous findings to probe the timescales over which these dynamics occur. For the case of UV-pump XUV-probe (h$\nu$=63 eV), we put specific emphasis on an enhanced level of neutral dissociation via the excited states $^3{Q_0}$ and $^1{Q_1}$^36^ by using a moderate UV intensity of 0.4 $\times 10^{12}$ W/$\hbox {cm}^2$. In contrast, for the NIR-pump XUV-probe (h$\nu$=75 eV) case, a relatively high NIR intensity of 4.3 $\times 10^{14}$ W/$\hbox {cm}^2$ was employed in order to ensure a high number of singly-charged iodine fragments that can be probed by the XUV pulses. Note that this value lies even slightly beyond the presented laser-intensity scan in Fig. 6. Figure 7 shows mass spectra resulting from the two-color experiments using either OL-laser pulses only (orange) or XUV pulses only (gray) as well as laser-early and laser-late cases (red and black, respectively). For the mass spectra involving XUV pulses, the pulse energy fluctuation was corrected on a shot-to-shot basis via the GMD data.

The UV-pump XUV-probe mass spectra, presented in Fig. 7a show that the total yield of the ionic fragments stemming from UV ionization (depicted in orange) is much lower compared to that generated by XUV pulses (shown in gray). Notably, and consistent with the deliberately adjusted low-intensity UV pulses, almost no charge states of iodine and only a limited number of ionic channels, primarily encompassing the parent, $\hbox {C}_5$ $\hbox {H}_{11}$, and alkyl groups, are generated. Note that for low *m*/*z* values, ringing of the Behlke switches overlaps the mass spectra. The XUV photons with an energy of 63 eV

lead to a significant formation of $\hbox {I}^{+}$, $\hbox {I}^{2+}$, and $\hbox {I}^{3+}$ even at the employed low intensity. Since this photon energy isn’t sufficient for the core ionization of ionic iodine, neither within the molecule nor as an atomic cation, double Auger-Meitner relaxation is expected to be the most prominent origin for these contributions^37^, but also excited states in the cation and/or charge transfer mechanisms from other sites of the molecular system can contribute to these charge states. As can be seen in Fig. 7a, for the UV-early case, the yield of singly-charged iodine is significantly lower than for the UV-late case, which indicates that the FEL produces excited states that allow the UV to finally ionize the iodine.

Pump-probe features can be seen for $\hbox {I}^{2+}$ in the mass spectrum for the UV-early case (see red mass spectrum in Fig. 7a). This signal predominantly results from the discussed neutral one-photon dissociation of the molecule^38–41^ into iodine and the alkyl group. This is followed by the ionization of the iodine atom via the XUV pulses and a subsequent Auger-Meitner decay which leads to the creation of $\hbox {I}^{2+}$ with $\hbox {I}^*$ in spin-orbit excited states:

1 $$\begin{aligned} \begin{aligned}&\textrm{C}_\textrm{5}\textrm{H}_{11}\textrm{I} + \textrm{UV} \rightarrow \textrm{C}_\textrm{5}\textrm{H}_{11} + \textrm{I}^{(*)} \\&\quad \textrm{C}_\textrm{5}\textrm{H}_{11} + \textrm{I}^{(*)} + \textrm{XUV} \rightarrow \textrm{C}_\textrm{5}\textrm{H}_{11} + \textrm{I}^{2+} \end{aligned} \end{aligned}$$

At larger internuclear distances, the charge transfer between the fragments becomes impossible, and the charge vacancy cannot be distributed between the atomic iodine and $\hbox {C}_5$ $\hbox {H}_{11}$, thus the evolution of the time-dependent $\hbox {I}^{2+}$ yield can be an indication for the closing of the charge-transfer channel^5^. The same effect can be identified for the delay-dependent peak at *m*/*z* = 42.3, representing the $\hbox {I}^{3+}$ fragment, as expected from a double-Auger-Meitner yield in the order of 15$\%$^37,42^.

The mass spectra represented by the black lines display molecules that first interact with the XUV pulses and then with the 500 fs delayed UV pulses (OL-late). The UV pulses appear to ionize a variety of excited molecular fragments generated by the initial XUV pulses, as it was concluded for the iodine above.

For the NIR-laser pulses the pump-probe mass spectra shown in Fig. 7b are fundamentally different, mainly due to the much higher intensity of the NIR pump-laser pulses, and their inability to resonantly excite the molecule through single-photon absorption. The multiphoton dissociative ionization results in several ionic fragments (orange), as well the iodine charge state $\hbox {I}^{+}$. ’NIR-early’ peaks can be seen in the mass spectrum for the case of a 2 ps delay (shown in red). Compared to the previously discussed UV-case the same peaks at *m*/*z* = 63.5 ($\hbox {I}^{2+}$) and *m*/*z* = 42.3 ($\hbox {I}^{3+}$) can be identified, with similar abundance for the former and an increased strength for the latter. $\hbox {I}^{2+}$ fragments appear less prominent, due to the lower number of neutral iodine created via the relatively strong NIR pulses (see also discussion below for the time-resolving scans over a longer delay range with multiple steps). In contrast, the additional $\hbox {I}^{+}$ fragments created by the NIR pulses, lead to a more prominent pump-probe signal at the $\hbox {I}^{3+}$ fragment as a consequence of the higher XUV photon energy in this part of the experiment, which is sufficient for core-ionizing singly charged iodine. The observed pump-probe signal in the $\hbox {I}^{4+}$ yield is consistent with the mentioned double-Auger yield. Residual Xe from a previous calibration is annotated in the inset, also showing a small pump-probe effect as can be expected from similar mechanisms.

**Fig. 8:**
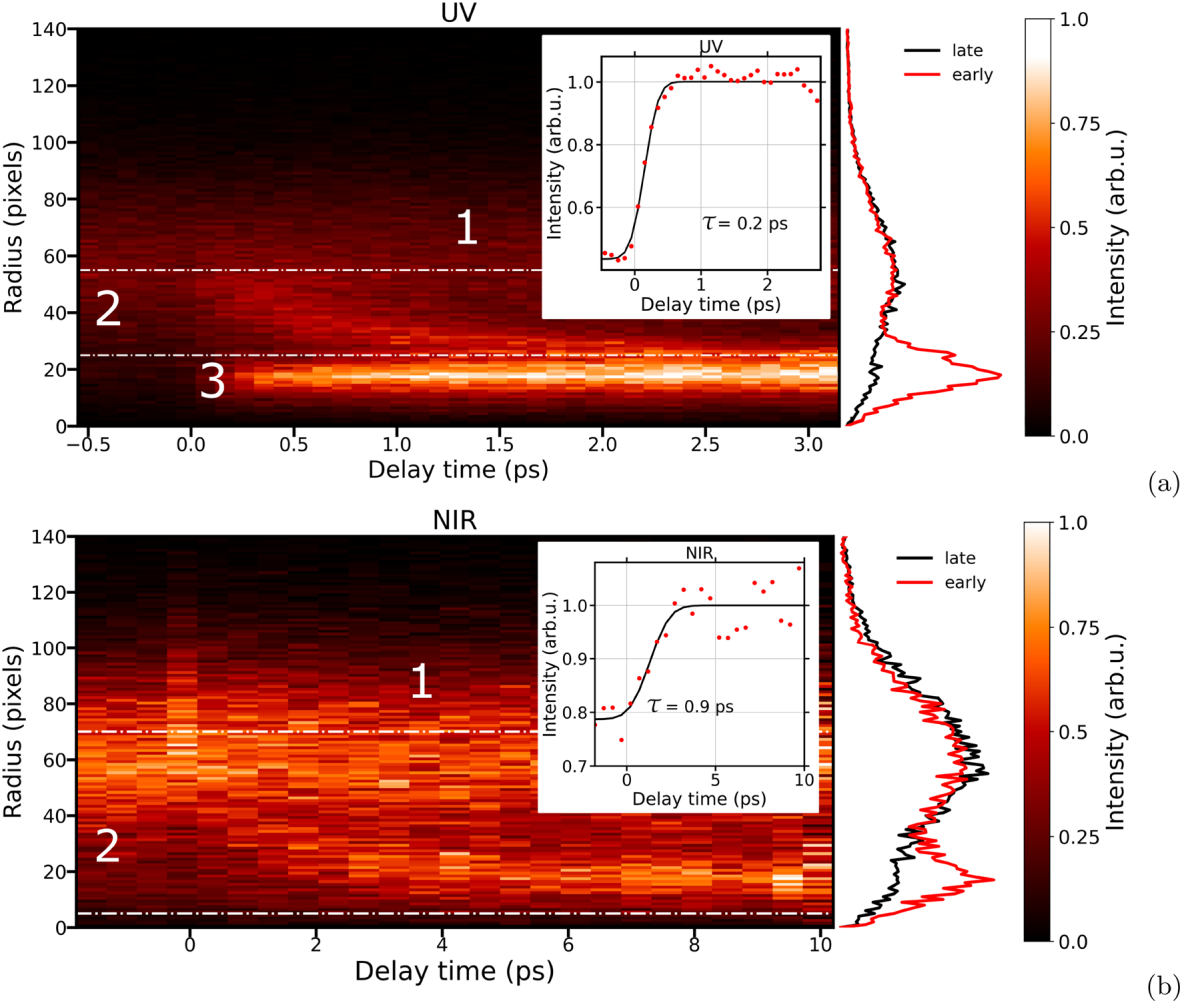
Radial distribution maps for the delay-dependent kinetic-energy distributions of $\hbox {I}^{2+}$: (**a**) UV pump—XUV probe and (**b**) NIR pump—XUV probe. The projections of the radial distributions are displayed next to the maps: UV/NIR late in black for delays − 0.25/− 1 ps and UV/NIR early in red for delays +2/+5 ps. Three regions are marked, named 1, 2 and 3. The insets represent the integrated yield of the ion-energy channel $\hbox {I}^{2+}$ within the dashed-dotted white lines capturing region 2 as a function of pump-probe delay (red points) with a fit to a normal cumulative distribution function (black line). The central value of the fitted Gaussian function is indicated as $\tau$.

Delay-dependent effects can be studied in more detail by investigating the kinetic energy (KE) of the relevant ionic fragments such as $\hbox {I}^{2+}$ as a function of pump-probe delay, see Fig. 8a for UV and (b) for NIR pulses. This data has been recorded with the PImMS camera, whereas the above-presented mass spectra were recorded via capacitive outcoupling of the MCP currents. For the UV-induced fragmentation, a scan in steps of 100 fs, covering a 3.5 ps range starting at − 0.5 ps and ending at 3 ps, is presented. The covered delay range for the NIR-case was 12 ps, ranging from − 2 ps to 10 ps, with scan steps of 500 fs since the observable processes cover a longer range than in the UV-case. Positive delays correspond to the OL-early and negative delays to OL-late regime.

A projection of the kinetic-energy dependent yields is plotted for the OL-early regime (red) and one for the OL-late (black) regime vertically between the radial distribution map and intensity color bar. The y-axis represents the radial distribution in pixels of the measured $\hbox {I}^{2+}$ fragments, which was derived from the velocity-map images from the PImMS camera. The radius is proportional to the magnitude of the initial ion velocity in the detector plane^30^. This representation of the data in Fig. 8 was chosen due to inability to perform high-quality inverse Abel transformation of the data (to yield the underlying three-dimensional velocity distribution) due to lack of statistics, particularly in the NIR-pump XUV-probe data.

In these delay-dependent ion velocity distributions, three distinct regions of signal can be identified, which were marked as Region 1, 2 and 3. Region 1 comprises a broad, high velocity ($\approx$ 30 to 100 pixels, corresponding to KEs between 1 and 3 eV), feature which is present over all pump-probe delays. This feature originates from XUV-only Coulomb explosion of the molecule to produce mutually repelling $\hbox {I}^{2+}$ and ionic alkyl cofragment(s). Given the propensity for single-photon XUV ionization to yield a doubly charged cation (which cannot Coulomb explode into $\hbox {I}^{2+}$ and other charged fragments), it is believed that much of this Coulomb explosion signal arises from absorption of multiple XUV photons. This process is more likely for the experiments with the NIR-pump pulse, owing to the higher XUV intensity employed.

Two time-dependent features are shown in Fig. 8, labeled 2 and 3 respectively, both of which appear shortly after time-zero. Time-zero is defined according to the method discussed in detail in^10^. The former feature has a delay-dependent KE, decreasing at longer pump-probe delay. In contrast, Region 3 comprises a signal with a constant low KE. As observed in previous OL-pump XUV-probe experiments, Region 2 is indicative of a Coulomb repulsion between fragments that is prompted by the XUV probe pulse^5,33–35,43^. At longer pump-probe delays, the fragments are at greater separations, and thus this Coulomb repulsion decreases. Region 3, which is only clearly observed in the case of UV photoexcitation, arises when $\hbox {I}^{2+}$ is produced by the XUV pulse in the absence of any charged co-fragments. This is indicative of a neutral photodissociation prompted by the optical laser, followed by XUV-ionization solely at the dissociated, isolated, iodine atom (see also Eq. 1). The KE distribution of the detected $\hbox {I}^{2+}$ ions (with a mean KE of $\approx$ 0.25 eV) reflects this photodissociation process to predominantly yield spin-orbit excited I* photoproducts, and is consistent with previous measurements^10,36^. In the case of NIR excitation, Region 3 signal could arise from a multi-photon induced neutral photodissociation to yield $\hbox {C}_5$ $\hbox {H}_{11}$+I followed by 4d ionization of the I photoproduct or from dissociative ionization to $\hbox {C}_5$ $\hbox {H}_{11}$+$\hbox {I}^+$ followed by valence ionization of the $\hbox {I}^+$ photoproduct. The lack of a clear Region 3 signal in the NIR experiment reflects the low likelihood of these processes.

**Fig. 9:**
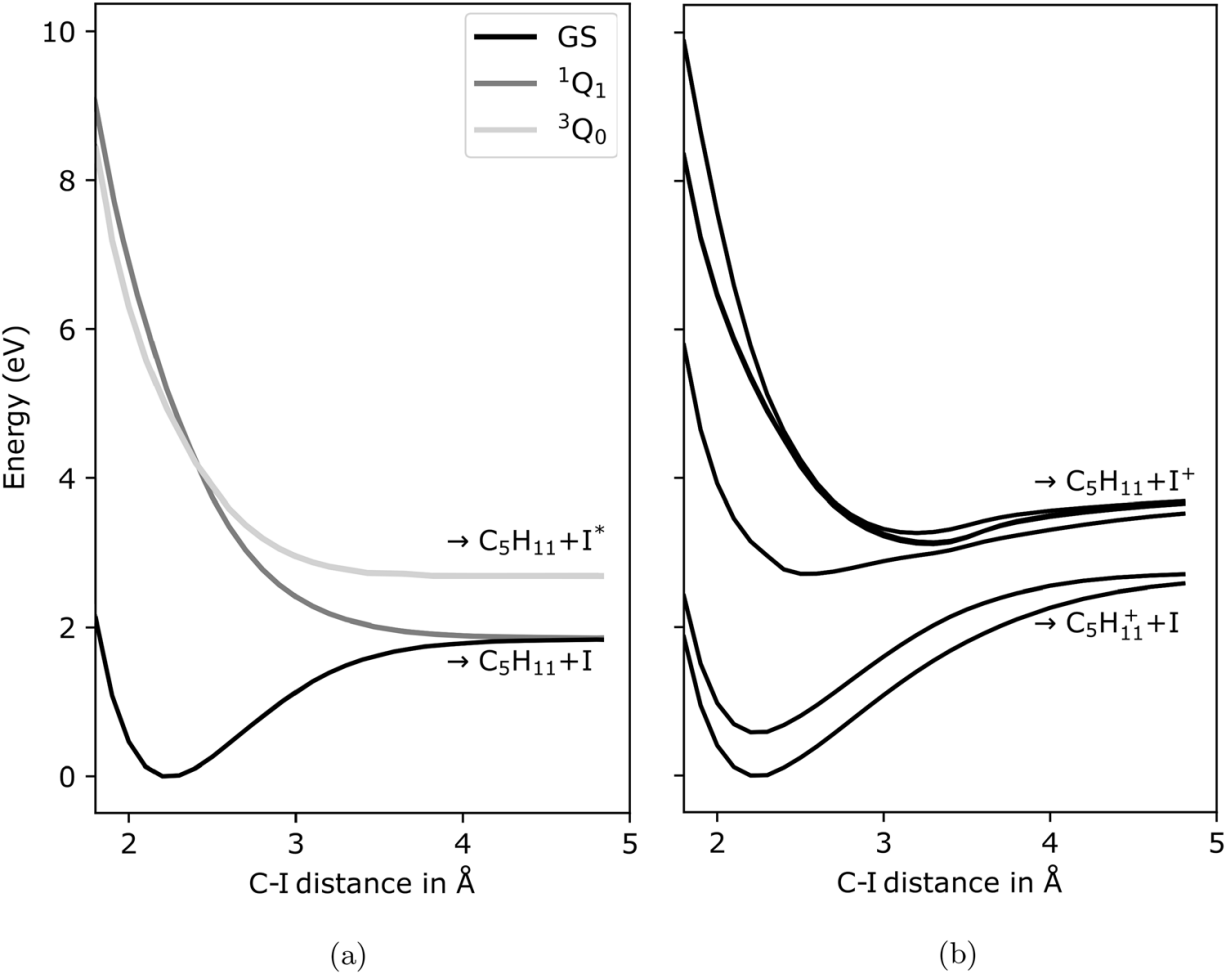
(**a**) Potential energy curves along the C–I bond for the ground state and the relevant $^3{Q_0}$ and $^1{Q_1}$ excited states. (**b**) Potential energy curves along the C–I bond for the energetically lowest states of the cation.

As mentioned above, the delay-dependent Coulomb repulsion contribution to the Region 2 signal indicates two mutually repelling charges following interaction with the XUV-probe pulse. In the case of UV photoexcitation, which is believed to predominantly induce single-photon neutral dissociation under the experimental conditions, this Coulomb repulsion can be induced by XUV-based valence ionization of the $\hbox {C}_5$ $\hbox {H}_{11}$ fragment alongside 4d ionization of the neutrally dissociated iodine (see Eq. 2).

2 $$\begin{aligned} \begin{aligned}&\textrm{C}_\textrm{5}\textrm{H}_{11}\textrm{I} + \textrm{UV} \rightarrow \textrm{C}_\textrm{5}\textrm{H}_{11} + \textrm{I}^{(*)} \\&\quad \textrm{C}_\textrm{5}\textrm{H}_{11} + \textrm{I}^{(*)} + \textrm{XUV} \rightarrow \textrm{C}_\textrm{5}\textrm{H}_{11}^{+} + \textrm{I}^{2+} \end{aligned} \end{aligned}$$

Minor contributions to this signal may also arise from multiphoton dissociative ionization to yield a charged alkyl fragment and a neutral iodine atom which is subsequently core ionized.

In the case of NIR excitation, which predominantly photoionizes the molecule under the given conditions, this signal is believed to mainly originate from such dissociative ionization processes (see Eq. 3).

3 $$\begin{aligned} \begin{aligned}&\textrm{C}_\textrm{5}\textrm{H}_{11}\textrm{I} + \textrm{NIR} \rightarrow \textrm{C}_\textrm{5}\textrm{H}_{11}^{+} + \textrm{I}\\&\quad \textrm{C}_\textrm{5}\textrm{H}_{11}^{+} + \textrm{I} + \textrm{XUV} \rightarrow \textrm{C}_\textrm{5}\textrm{H}_{11}^{+} + \textrm{I}^{2+} \end{aligned} \end{aligned}$$

We note that a detailed modeling of the time-evolving KE distribution associated with Region 2 is challenging in the present experiment due to uncertainty in the identity of the charged alkyl cofragment and the trajectories of any neutral fragments produced simultaneously. As seen in the mass spectra reported in Fig. 7, the NIR pulse produces a range of alkyl ions. Whilst UV excitation will yield predominantly $\hbox {C}_5$ $\hbox {H}_{11}$ fragments, the subsequent XUV ionization may yield smaller daughter ions. In principle, if the relative three-dimensional momentum vectors of the $\hbox {I}^{2+}$ ion and repelling partner ions could be determined (by using coincidence or covariance analysis), additional insights into the OL-induced fragmentation dynamics could be obtained. In the present work, this was not possible, due to limited statistics/data volume, and the time resolution of the PImMS camera which precluded three-dimensional velocity-map imaging.

To examine the timescales of the underlying processes leading to Region 2 signal in the two excitation regimes, the integrated ion intensities of velocity ranges of interest are shown as a function of pump-probe delay in the insets of Fig. 8a,b. We note that, particularly in the NIR case, the chosen region of interest includes significant over-lap from XUV-only signal (i.e., channel 2) at higher radii. This is to ensure that all signal coming from the delay-dependent feature is captured. To quantify these timescales, the delay-dependent intensities were fitted with a Gaussian cumulative distribution. In the case of UV excitation, this signal rises on a timescale of a few hundred femtoseconds ($\tau =200$ fs), close to the estimated temporal resolution of the experiment. In the case of UV excitation, a velocity range was chosen which excludes Region 3. A similar rise time as in earlier studies is observed for the lower velocity, Region 3 signal^10^. In contrast, for NIR excitation, the timescale for the formation of Region 2 signal is several times slower ($\tau =900$ fs), implying that there is a substantial time between interaction with the NIR pulse and the ultimate fragmentation.

The calculated potential energy curves (PECs) shown in Fig. 9 suggest a reason for the strong difference in time scales. For the neutral molecule, a state-averaged complete active space (SA-CASSCF) calculation was conducted using an orbital space of four orbitals with six electrons and averaging over the three lowest singlet states employing the 6-311G(d,p) basis set^11,44^. From the obtained set of orbitals, the basis states for the spin-orbit coupling (SOC) calculations were constructed consisting of the six lowest triplet and four lowest singlet states were obtained by diagonalizing the configuration interaction (CI) matrix in this active space. The Breit–Pauli Hamiltonian was then diagonalized in these basis states. The PECs of the $^3{Q_0}$ and $^1{Q_1}$ excited states of the neutral molecule, which are populated by the UV pulses, are dissociative along the C-I coordinate (Fig. 9a) and lead to very prompt photodissociation.

For the molecular cation mainly addressed by NIR pump pulses, a SA-CASSCF calculation involving 6 orbitals and 5 electrons was performed, state averaged over 6 doublet states, and employing the same atomic orbital basis set as for the neutral case. The Breit–Pauli Hamiltonian was diagonalized considering the 4 lowest quadruplet and 6 lowest doublet states. The calculations were performed with Molpro version 2020.1^45^ and the results are depicted in Fig. 9b. The lowest PECs for the cation, which are likely to be dominantly populated by the strong-field NIR pulse, have potential wells along this coordinate, these states are predicted to be non-dissociative (Fig. 9b). This suggests that the dissociation triggered by the NIR pulse proceeds along a different reaction coordinate(s), probably via more complex structural rearrangements that take significantly longer to evolve.

## Conclusions

The OL-induced dissociation of the prototypical chiral molecule 1-iodo-2-methyl-butane was studied through measuring intensity-dependent fragment yields for femtosecond UV (267 nm) and NIR (800 nm) laser pulses. These photoinduced dynamics were also probed via femtosecond time-resolved inner-shell photoionization at the I 4d edge using XUV pulses from FLASH in conjuction with velocity-map imaging. By analyzing the KE distribution of the $\hbox {I}^{2+}$ as a function of different delay times, the dynamics of the neutral and ionic dissociation channels populated by the UV and NIR pulses were explored. Under the employed conditions, the UV pulses predominantly led to resonant single-photon absorption whilst the NIR pulse ionized the molecule, and could initiate dissociative ionization. These dissociative ionization process was found to proceed several times slower than the UV-induced photodissociation. This observation could be rationalized in terms of the weakly bound nature of low-lying PECs of the cation along the C–I stretch coordinate.

For this experiment, observing time-resolved chiral signals (e.g. PECD) was out of reach. We however note that the future for such work at FLASH and also the European XFEL, both in Germany, is very bright. Key technical upgrades on optical laser capabilities, polarization control, seeding, and short-pulse operation will enable new paths for chirality research^46,47^.

## Data Availability

The datasets used and analyzed during the current study are available from the corresponding author upon reasonable request.

## References

[1] Young, L. et al. Roadmap of ultrafast X-ray atomic and molecular physics. *J. Phys. B At. Mol. Opt. Phys.* **51**, 032003 (2018).

[2] McNeil, B. & Thompson, N. X-ray free-electron lasers. *Nat. Photon.* **4**, 814–821 (2010).

[3] Emma, P. et al. First lasing and operation of an ångstrom-wavelength free-electron laser. *Nat. Photon.* **4**, 641–647 (2010).

[4] Hartmann, G. et al. Attosecond time–energy structure of X-ray free-electron laser pulses. *Nat. Photon.* **12**, 215–220 (2018).

[5] Erk, B. et al. Imaging charge transfer in iodomethane upon X-ray photoabsorption. *Science* **345**, 288 (2014). 25035485 10.1126/science.1253607

[6] Motomura, K. et al. Charge and nuclear dynamics induced by deep inner-shell multiphoton ionization of I molecules by intense X-ray free-electron laser pulses. *J. Phys. Chem. Lett.* **6**, 2944–2949 (2015). 26267186 10.1021/acs.jpclett.5b01205

[7] Mertens, K. et al. Soft X-ray multiphoton excitation of small iodine methane derivatives. *J. Mod. Opt.* **63**, 383 (2016).

[8] Köckert, H. et al. UV-induced dissociation of CH2Br I probed by intense femtosecond XUV pulses. *J. Phys. B: At. Mol. Opt. Phys.* **55**, 014001 (2022).

[9] Freitas, J. E. & El-Sayed, M. A. The wavelength dependence of the rates of internal energy redistribution during the photodissociation of 3-iodopyridine. *J. Phys. Chem.* **99**, 7395–7406 (1994).

[10] Allum, F. et al. A localized view on molecular dissociation via electron-ion partial covariance. *Comm. Chem.* **5**, 42 (2022). 10.1038/s42004-022-00656-w PMC9814695 36697752

[11] Krishnan, R. et al. Self-consistent molecular orbital methods. XX. A basis set for correlated wave functions. *J. Chem. Phys.* **72**, 650–654 (1980).

[12] Ritchie, B. Theory of the angular distribution of photoelectrons ejected from optically active molecules and molecular negative ions. *Phys. Rev. A* **13**, 1411 (1976).

[13] Powis, I. Photoelectron circular dichroism of the randomly oriented chiral molecules glyceraldehyde and lactic acid. *J. Chem. Phys.* **112**, 301 (2000).

[14] Böwering, N. et al. Asymmetry in photoelectron emission from chiral molecules induced by circularly polarized light. *Phys. Rev. Lett.* **86**, 1187 (2001). 11178040 10.1103/PhysRevLett.86.1187

[15] Ilchen, M. et al. Emitter-site-selective photoelectron circular dichroism of trifluoromethyloxirane. *Phys. Rev. A* **95**, 053423 (2017).

[16] Ilchen, M. et al. Site-specific interrogation of an ionic chiral fragment during photolysis using an X-ray free-electron laser. *Comm. Chem.* **4**, 119 (2021). 10.1038/s42004-021-00555-6 PMC9814667 36697819

[17] Ackermann, W. et al. Operation of a free-electron laser from the extreme ultraviolet to the water window. *Nat. Photon* **1**, 336–342 (2007).

[18] Feldhaus, J. FLASH-the first soft X-ray free electron laser (FEL) user facility. *Phys. B At. Mol. Opt. Phys.* **43**, 194002 (2010).

[19] Erk, B. et al. CAMP@FLASH: an end-station for imaging, electron- and ion spectroscopy, and pump-probe experiments at the FLASH free-electron laser. *J. Synchrotron Radiat.* **25**, 1529–1540 (2018). 30179194 10.1107/S1600577518008585 PMC6140390

[20] Domondon, A. T. & Tong, X. M. Photoabsorption spectra of I and its ions in the 4d region. *Phys. Rev. A* **65**, 032718 (2002).

[21] Kjeldsen, H. et al. Absolute photoionization cross sections of and in the 4d ionization region. *Phys. Rev. A* **62**, 020702 (2000).

[22] Moulton, P. F. Spectroscopic and laser characteristics of Ti:Al2O3. *J. Opt. Soc. Am. B* **3**, 125–133 (1986).

[23] Nikogosyan, D. N. Beta barium borate (BBO). *Appl. Phys. A* **52**, 359–368 (1991).

[24] von Korff Schmising, C. et al. Generating circularly polarized radiation in the extreme ultraviolet spectral range at the free-electron laser FLASH. *Rev. Sci. Instrum.* **88**, 053903 (2017). 10.1063/1.4983056 28571434

[25] Grimm, O., et al. Longitudinal bunch shape diagnostics with coherent radiation and a transverse deflecting cavity at TTF2. In *SLAC National Accelerator Lab., Menlo Park, CA (USA)*, *No. SLAC-PUB-11387* (2005).

[26] Röhrs, M., et al., Slice emittance measurements at FLASH. In *Proc. 10th Eur. Particle Accelerator Conference, Edinburgh, Scotland, (UK)*, 77 (2006).

[27] Tiedtke, K. et al. Gas detectors for X-ray lasers. *J. Appl. Phys.* **103**, 094511 (2008).

[28] Löhl, F. et al. Electron bunch timing with femtosecond precision in a superconducting free-electron laser. *Phys. Rev. Lett.* **104**, 144801 (2010). 20481941 10.1103/PhysRevLett.104.144801

[29] Klein, S. M., Zhang, C. & Jiang, Y. L. Simple synthesis of fresh alkyl iodides using alcohols and hydriodic acid. *Tetrahedron Lett.* **49**, 2638–2641 (2008).

[30] Eppink, A. T. J. B. & Parker, D. H. Velocity map imaging of ions and electrons using electrostatic lenses: application in photoelectron and photofragment ion imaging of molecular oxygen. *Rev. Sci. Instrum.* **68**, 3477–3484 (1997).

[31] Nomerotski, A. et al., Pixel imaging mass spectrometry with fast silicon detectors. *Nucl. Instrum. Methods Phys. Res. Sect. A*. **633**, S243–S246 (2011).

[32] Redlin, H. et al. The FLASH pump–probe laser system: Setup, characterization and optical beamlines. *Nucl. Instrum. Methods Phys. Res. A* **635**, S88–S93 (2011).

[33] Allum, F. et al. Direct momentum imaging of charge transfer following site-selective ionization. *Phys. Rev. A* **108**, 043113 (2023).

[34] Boll, R. et al. Charge transfer in dissociating iodomethane and fluoromethane molecules ionized by intense femtosecond X-ray pulses. *Struct. Dyn.* **3**, 043207 (2016). 27051675 10.1063/1.4944344 PMC4808069

[35] Amini, K. et al. Photodissociation of aligned I and I molecules probed with time-resolved Coulomb explosion imaging by site-selective extreme ultraviolet ionization. *Struct. Dyn.* **5**, 014301 (2018). 29430482 10.1063/1.4998648 PMC5785297

[36] Corrales, M. E. et al. Structural dynamics effects on the ultrafast chemical bond cleavage of a photodissociation reaction. *Phys. Chem. Chem. Phys.* **16**, 8812–8818 (2014). 24418888 10.1039/c3cp54677b

[37] Eland, J. H. D. et al. Ion charge-resolved branching in decay of inner shell holes in Xe up to 1200 eV. *J. Phys. B: At. Mol. Opt. Phys.* **48**, 205001 (2015).

[38] Downes-Ward, B. et al. Photodissociation dynamics of methyl iodide across the A-band probed by femtosecond extreme ultraviolet photoelectron spectroscopy. *J. Phys. B. Mol. Opt. Phys.* **54**, 134003 (2021). 10.1039/d0cp03478a 33146165

[39] Boschi, R. & Salahub, D. The far ultra-violet spectra of some 1-iodoalkanes. *Mol. Phys.* **24**, 289–299 (1972).

[40] Todt, M. A. et al. Subpicosecond HI elimination in the 266 nm photodissociation of branched iodoalkanes. *Phys. Chem. Chem. Phys.* **22**, 27338–27347 (2020). 33231219 10.1039/c9cp06460e

[41] Ross, P. L. & Johnston, M. V. Excited state photochemistry of iodoalkanes. *J. Phys. Chem.* **99**, 4078–4085 (1995).

[42] Forbes, et al. Photoionization of the I 4d and valence orbitals of methyl iodide. *J. Phys. B: At. Mol. Opt. Phys.* **53**, 155101 (2020).

[43] Allum, F. et al. Multi-channel photodissociation and XUV-induced charge transfer dynamics in strong-field-ionized methyl iodide studied with time-resolved recoil-frame covariance imaging. *Faraday Discuss.* **228**, 571–596 (2020). 10.1039/d0fd00115e 33629700

[44] Glukhovtsev, M. N. et al. Extension of Gaussian-2 (G2) theory to bromine- and iodine-containing molecules: Use of effective core potentials. *J. Chem. Phys.* **103**, 1878–1885 (1995).

[45] Werner, H.-J. et al. Molpro: a general-purpose quantum chemistry program package. *WIREs Comput. Mol. Sci.* **2**, 242–253 (2012).

[46] Beye, M. et al. FLASH and the FLASH2020+ project-current status and upgrades for the free-electron laser in Hamburg at DESY. *Eur. Phys. J. Plus* **138**, 193 (2023).

[47] Ilchen, M. et al. Opportunities for gas-phase science at short-wavelength free-electron lasers with undulator-based polarization control Phys. *Rev. Res.* **7**, 011001 (2025).

